# Supplementary material for: CYFIP1 governs the development of cortical axons by modulating calcium availability
Source: Nat Commun. 2025 Nov 28;16:10764. doi: 10.1038/s41467-025-65801-0 (PMC12663352; doi:10.1038/s41467-025-65801-0)
Supplement: Supplementary file 1 — Supplementary Information [file 41467_2025_65801_MOESM1_ESM.pdf]

Supplementary Information

CYFIP1 governs the development of cortical axons by modulating calcium availability

Ricci et al.

Fig. S1

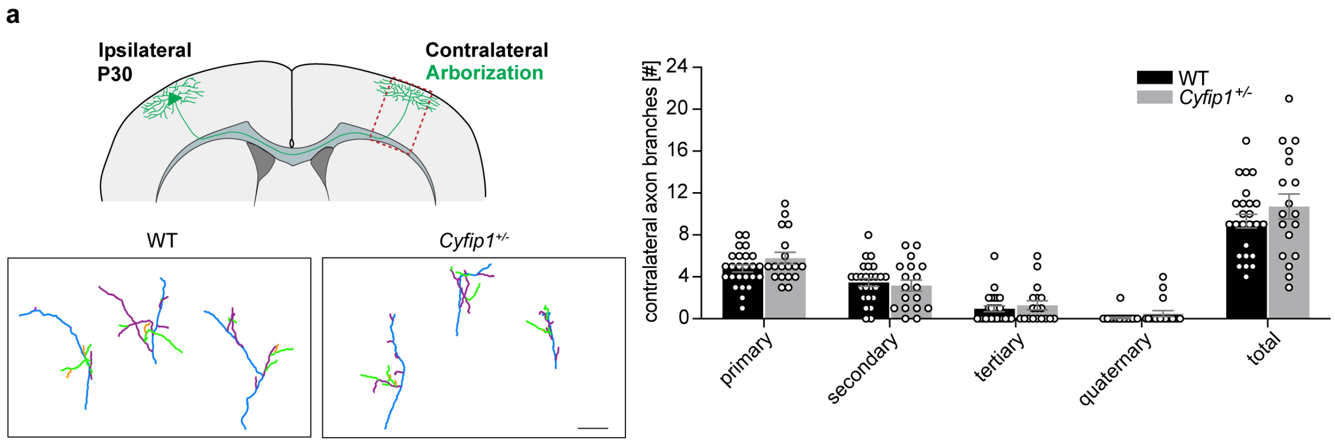

Fig. S1. Deficits in axonal arborization in *Cyfip1*$^{+/-}$ mice are transitory. a Upper left, representative schematic of a P30 brain slice after IUE with a tdTomato-expressing plasmid at E15.5. Lower left, representative axonal arborization traces of WT and *Cyfip1*$^{+/-}$ mice at P30 (main axonal branch, blue; primary branch, purple; secondary branch, green; tertiary branch, yellow). Scale bar 100 µm. Right, quantification of contralateral axonal branches in WT and *Cyfip1*$^{+/-}$ axons (WT n=25 axons from 4 mice; *Cyfip1*$^{+/-}$ n=18 axons from 3 mice) (mean ± SEM; Holm-Sidak t-test; primary: p=0.4411; secondary: p=0.7689; tertiary: p=0.7689; quaternary: p=0.4411; total: p=0.6273). Source data are provided as a Source Data file.

Fig. S2

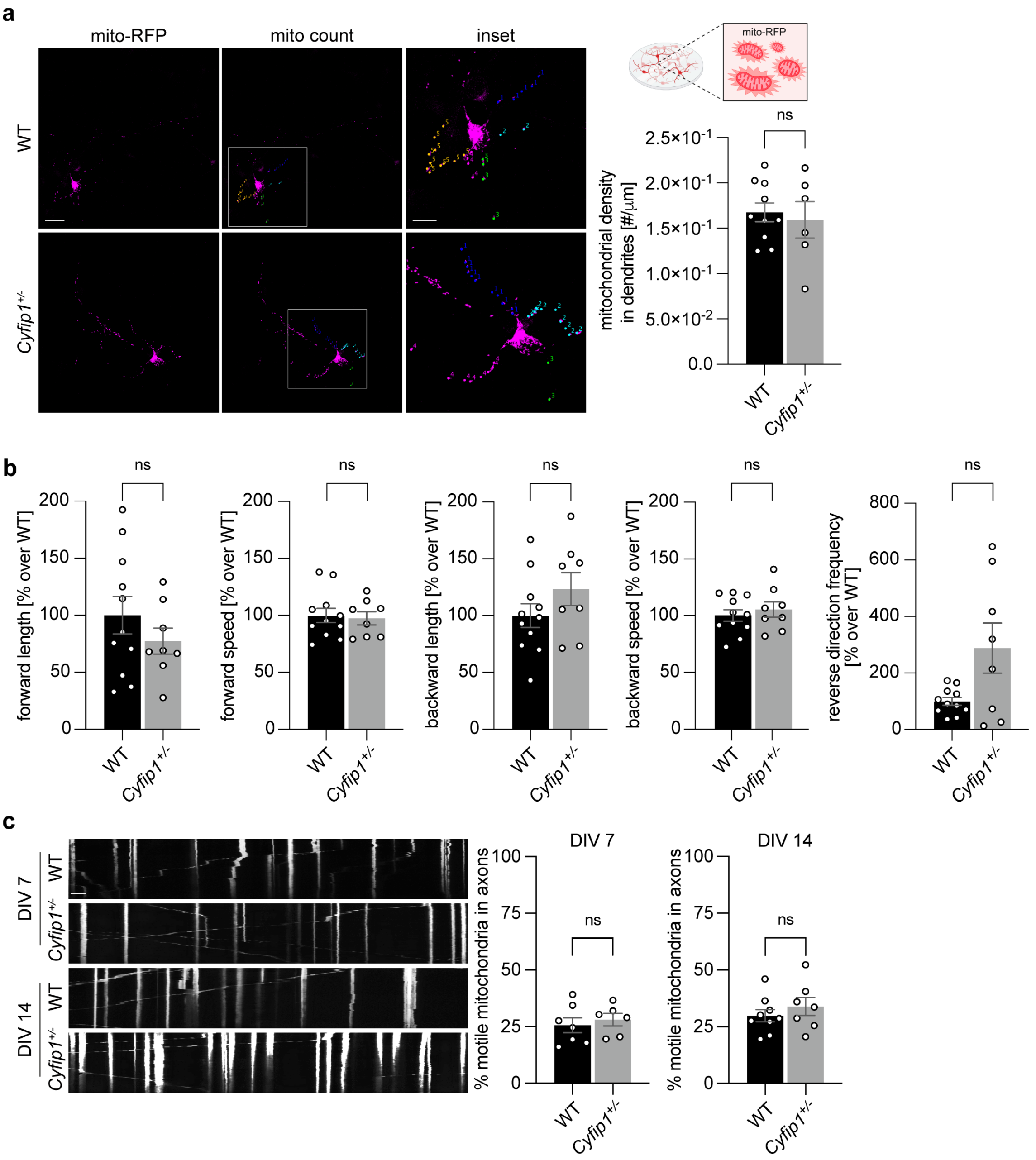

Fig. S2. Mitochondrial defects are restricted to axons and early developmental stages. **a** Left, representative images of DIV 3 WT and *Cyfip1*$^{+/-}$ cortical neurons after transfection with mito-RFP plasmid (magenta). The number of mitochondria along dendrites was calculated and normalized to the length of each dendrite. The average density of mitochondria per dendrite was determined for each neuron and then averaged by animal. Scale bar 20 μm. Inset scale bar 10 μm. Up right, illustration depicting the experimental set up (created in BioRender. https://BioRender.com/93y8ggs). Bottom right, average mitochondrial density along the entire neuron (WT n=10 embryo, *Cyfip1*$^{+/-}$ n=6 embryo;

mean ± SEM; two-tailed Mann-Whitney test; p=0.8749). **b** Representative histograms showing forward length, forward speed, backward length, backward speed and reverse direction frequency of mitochondria movement along axons of WT and *Cyfip1*$^{+/-}$ DIV 3 cortical neurons (WT n=11 embryos, 53 axons, *Cyfip1*$^{+/-}$ n=8 embryos, 31 axons; mean ± SEM; two-tailed Mann-Whitney test, forward length p=0.3511, forward speed p=0.9678, backward length p=0.2723, backward speed p=0.6574, reverse direction frequency p=0.2375). **c** Left, representative kymographs showing transport of mitochondria along axons in WT and *Cyfip1*$^{+/-}$ DIV 7 and DIV 14 cortical neurons. Scale bar 10 μm. Right, percentage of motile mitochondria in WT and *Cyfip1*$^{+/-}$ DIV 7 and DIV 14 cortical neurons (DIV 7: WT n=7 embryos, 33 axons, *Cyfip1*$^{+/-}$ n=6 embryos, 26 axons; mean ± SEM; two-tailed Mann-Whitney test, p=0.4452. DIV 14: WT n=9 embryos, 43 axons, *Cyfip1*$^{+/-}$ n=7 embryos, 31 axons; mean ± SEM; two-tailed Mann-Whitney test, p=0.4698). Source data are provided as a Source Data file.

Fig. S3

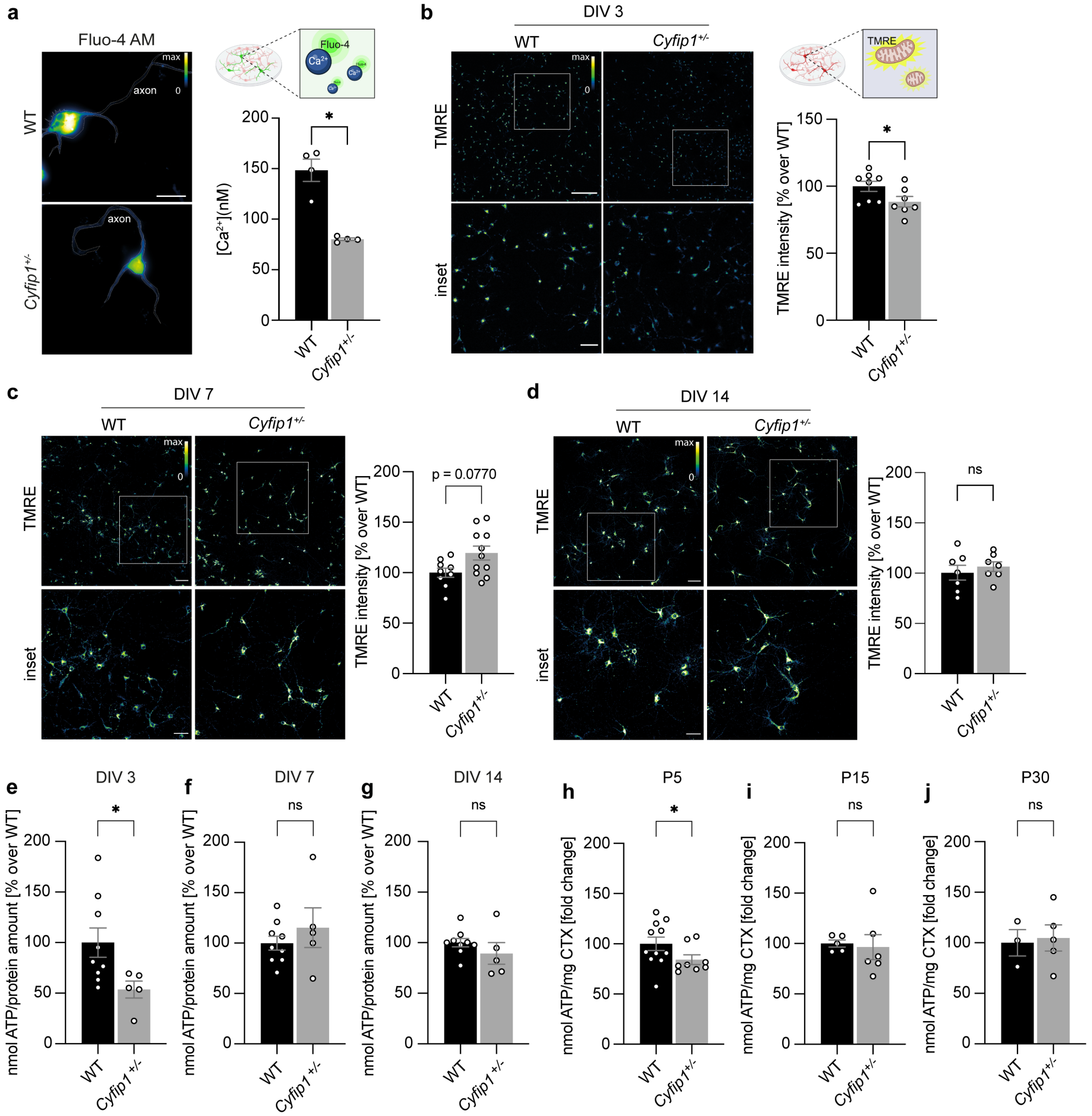

Fig. S3. Whole cell mitochondrial activity is normalized at later developmental stages. **a** Left, representative images of Fluo-4 AM intensity in the cell body of WT and *Cyfip1*$^{+/-}$ DIV 3 cortical neurons. Scale bar 20 μm. Up right, illustration depicting the experimental set up (created in BioRender. https://BioRender.com/cdkbr40). Bottom right, cytosolic calcium concentration in WT and *Cyfip1*$^{+/-}$ neurons. (WT n=4 embryos, 39 neurons, *Cyfip1*$^{+/-}$ n=4 embryos, 36 neurons; mean ± SEM; two-tailed Mann-Whitney test, p=0.0286). **b-d** Left, representative images of TMRE intensity in the cell body of WT and *Cyfip1*$^{+/-}$ cortical neurons

at DIV 3 (**b**), DIV 7 (**c**) and DIV 14 (**d**). Scale bar: DIV3, 200 μm, inset scale bar 50 μm, DIV7 and 14, 100 μm, inset scale bar 50 μm. Up right, illustration depicting the experimental set up (created in BioRender). Bottom right, histogram representing the average TMRE intensity in WT and *Cyfip1*$^{+/-}$ cell body (DIV 3, WT n=8 embryos, 12247 neurons, *Cyfip1*$^{+/-}$ n=7 embryos, 11286 neurons; mean ± SEM; two-tailed Mann-Whitney test, p=0.0401; DIV 7, WT n=9 embryos, 11660 neurons, *Cyfip1*$^{+/-}$ n=11 embryos, 9198 neurons, mean ± SEM, two-tailed Mann-Whitney test, p=0.0770; DIV 14, WT n=7 embryos, 3987 neurons, *Cyfip1*$^{+/-}$ n=7 embryos, 3652 neurons, mean ± SEM, two-tailed Mann-Whitney test, p=0.6439). **e-g** Histograms representing the quantification of ATP level in DIV 3 (**e**), DIV 7 (**f**), and DIV 14 (**g**) WT and *Cyfip1*$^{+/-}$ mouse primary cortical neurons, normalized on the protein concentration (DIV 3, WT n=9 biological replicates, *Cyfip1*$^{+/-}$ n=5 biological replicates, mean ± SEM, two-tailed Mann-Whitney test p=0.0120; DIV 7, WT n=9 biological replicates, *Cyfip1*$^{+/-}$ n=5 biological replicates, mean ± SEM, two-tailed Mann-Whitney test p=0.5185; DIV 14, WT n=9 biological replicates, *Cyfip1*$^{+/-}$ n=5 biological replicates, mean ± SEM, two-tailed Mann-Whitney test p=0.1898). **h-j** ATP level measured by fluorescence intensity. The histograms represent the quantification of ATP level in P5 (**h**), P15 (**i**), and P30 (**j**) WT and *Cyfip1*$^{+/-}$ mice cortex, normalized on the tissue's weight. (P5, WT n=11, *Cyfip1*$^{+/-}$ n=8, mean ± SEM, two-tailed Mann-Whitney test p=0.0328; P15, WT n=5, *Cyfip1*$^{+/-}$ n=6, mean ± SEM, two-tailed Mann-Whitney test p=0.4286; P30, WT n=3, *Cyfip1*$^{+/-}$ n=5, mean ± SEM, two-tailed Mann-Whitney test p>0.9999). Source data are provided as a Source Data file.

Fig. S4

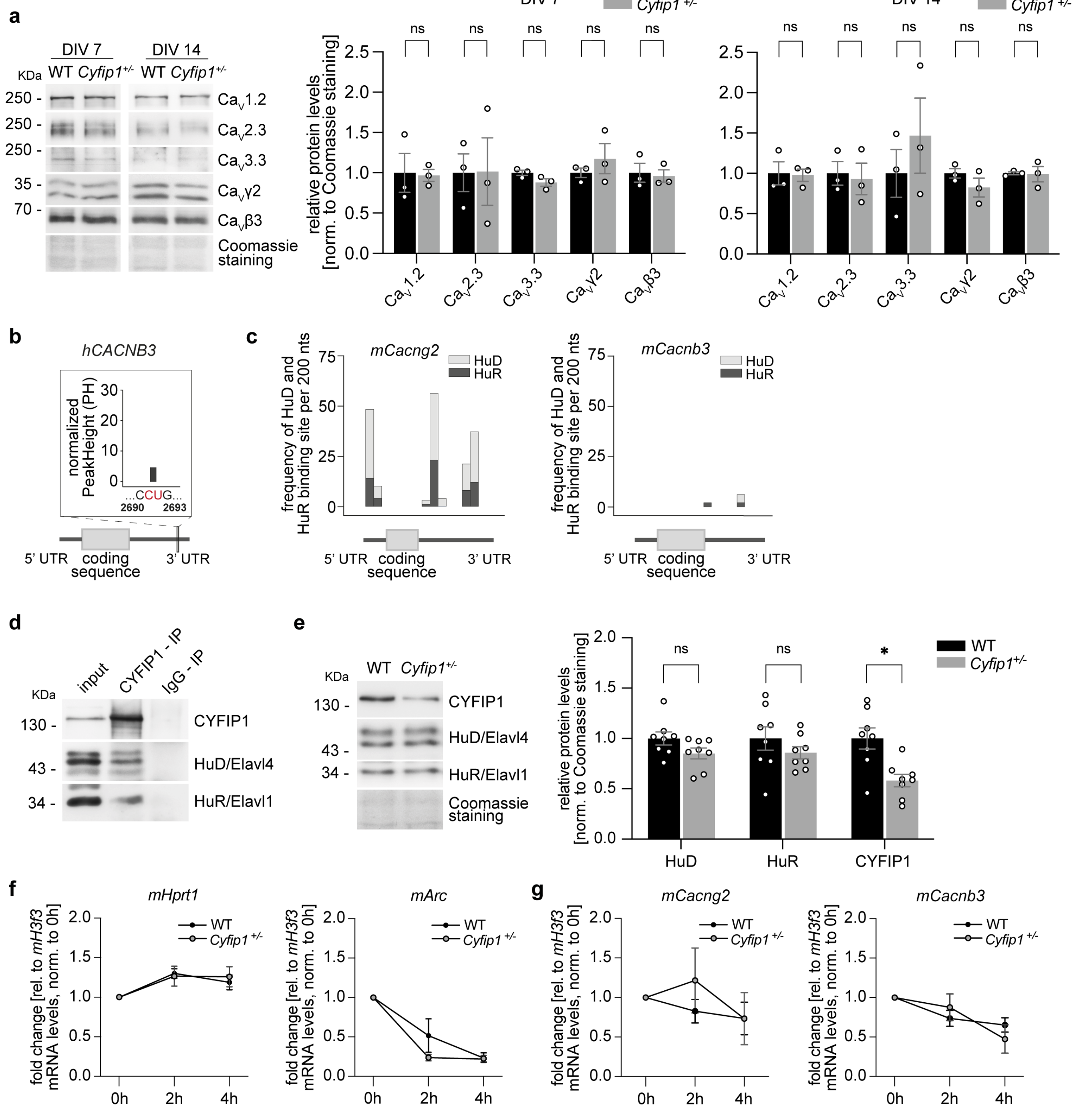

Fig. S4. CYFIP1 binds HuD and HuR but their protein expression in unaffected in *Cyfip1*$^{+/-}$ cortical neurons. a Left, representative Western Blot showing $Ca_V1.2$ (CACNA1C), $Ca_V2.3$ (CACNA1E), $Ca_V3.3$ (CACNA1I), $Ca_V\gamma2$ (CACNG2/Stargazin) and $Ca_V\beta3$ (CACNB3) in membrane-enriched fractions from WT and *Cyfip1*$^{+/-}$ DIV 7 and DIV 14 cortical neurons. The molecular weight of each protein is indicated in kDa. Right, histogram representing $Ca_V1.2$, $Ca_V2.3$, $Ca_V3.3$, $Ca_V\gamma2$ and $Ca_V\beta3$ protein expression levels in

membrane-enriched fractions from WT and *Cyfip1*$^{+/-}$ DIV 7 and DIV 14 cortical neurons. Protein levels were normalized to Coomassie staining and expressed as a fold change over WT (DIV 7: WT n=3 embryos, *Cyfip1*$^{+/-}$ n=3 embryos; mean ± SEM; Multiple unpaired two-tailed t-test, $Ca_V1.2$ p=0.9730, $Ca_V2.3$ p>0.9999, $Ca_V3.3$ p=0.6723, $Ca_V\gamma2$ p=0.8740, $Ca_V\beta3$ p>0.9999; DIV 14: WT n=3 embryos, *Cyfip1*$^{+/-}$ n=3 embryos; mean ± SEM; Multiple unpaired two-tailed t-test, $Ca_V1.2$ p>0.9999, $Ca_V2.3$ p>0.9999, $Ca_V3.3$ p=0.9919, $Ca_V\gamma2$ p=0.9222, $Ca_V\beta3$ p>0.9999). **b** Histogram representing the frequency of nELAVL binding sites on the 3' UTR of *hCACNB3* mRNAs as found in the published CLIP-seq data (Scheckel et al., 2016). **c** Histograms representing the frequency of predicted binding sites of HuD and HuR on the 5' UTR, coding sequence and 3'UTR of *mCacng2* and *mCacnb3* mRNAs, generated using RBPmap. **d** Representative Western blot of CYFIP1 protein immunoprecipitation from DIV3 WT cortical neurons, showing the detection of HuD/Elavl4 and HuR/Elavl1 proteins. **e** Left, representative Western Blot showing CYFIP1, HuD/Elavl4 and HuR/Elavl1 proteins in WT and *Cyfip1*$^{+/-}$ DIV 3 cortical neurons. The molecular weight of each protein is indicated in kDa. Right, histogram representing HuD/Elavl4, HuR/Elavl1 and CYFIP1 protein expression levels normalized to Coomassie staining and expressed as a fold change over WT (WT n=8 embryos, *Cyfip1*$^{+/-}$ n=8 embryos; mean ± SEM; Multiple two-tailed Mann-Whitney t-test, HuD/Elavl4 p=0.1828, HuR/Elavl1 p=0.2907, CYFIP1 p=0.0116). **f** Graph representing the mRNA decay of *mHprt1* and *mArc* after transcriptional shutdown with Actinomycin D in WT and *Cyfip1*$^{+/-}$ DIV 3 cortical neurons. mRNA expression levels were normalized to *mH3f3* mRNA at each time point (0, 2 and 4h after Actinomycin D treatment). (WT n=3 embryos, *Cyfip1*$^{+/-}$ n=4 embryos; mean ± SEM, *mHprt1*: Two-way ANOVA, $F_{(2,15)}$ = 0.1648, p=0.8496; time p=0.0210, genotype p=0.8737, interaction p=0.8496. *mArc*: Two-way ANOVA, $F_{(2,15)}$ = 1.924, p=0.1803; time p<0.0001, genotype p=0.1598, interaction p=0.1803). **g** Relative expression levels of *mCacng2* and *mCacnb3* mRNA at 0, 2 and 4h after transcriptional shutdown with Actinomycin D. mRNA expression levels were normalized to *mH3f3* mRNA at each time point (WT n=3 embryos, *Cyfip1*$^{+/-}$ n=3 embryos; mean ± SEM, *mCacng2*: Two-way ANOVA, $F_{(2,11)}$ = 0.4482, p=0.6490; time p=0.4312, genotype p=0.5201, interaction p=0.6490; *mCacnb3*: Two-way ANOVA, $F_{(2,12)}$ = 0.9701, p=0.4069; time p=0.0086, genotype p=0.8984, interaction p=0.4069). Source data are provided as a Source Data file.

Fig. S5

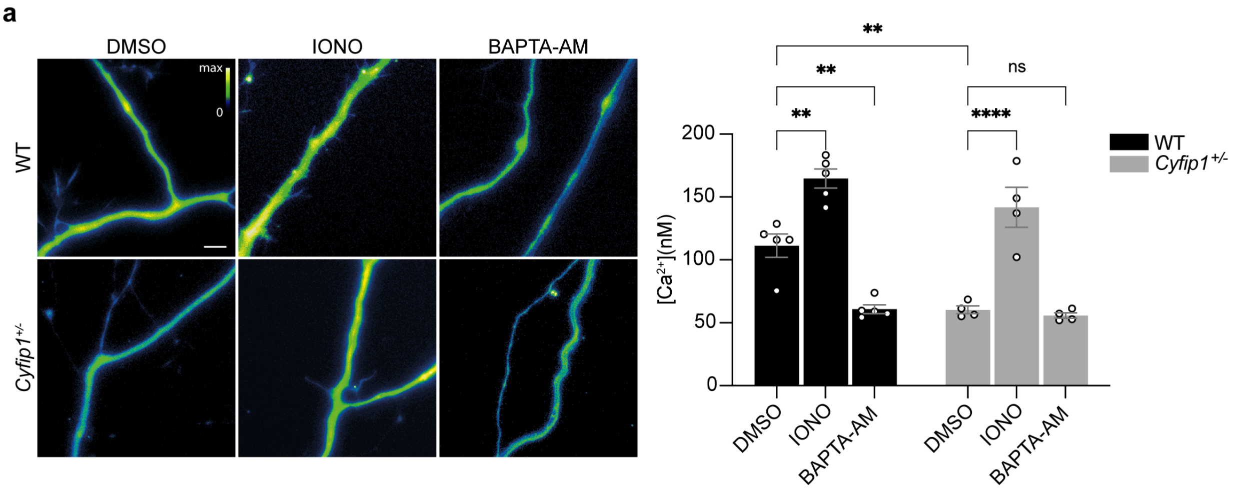

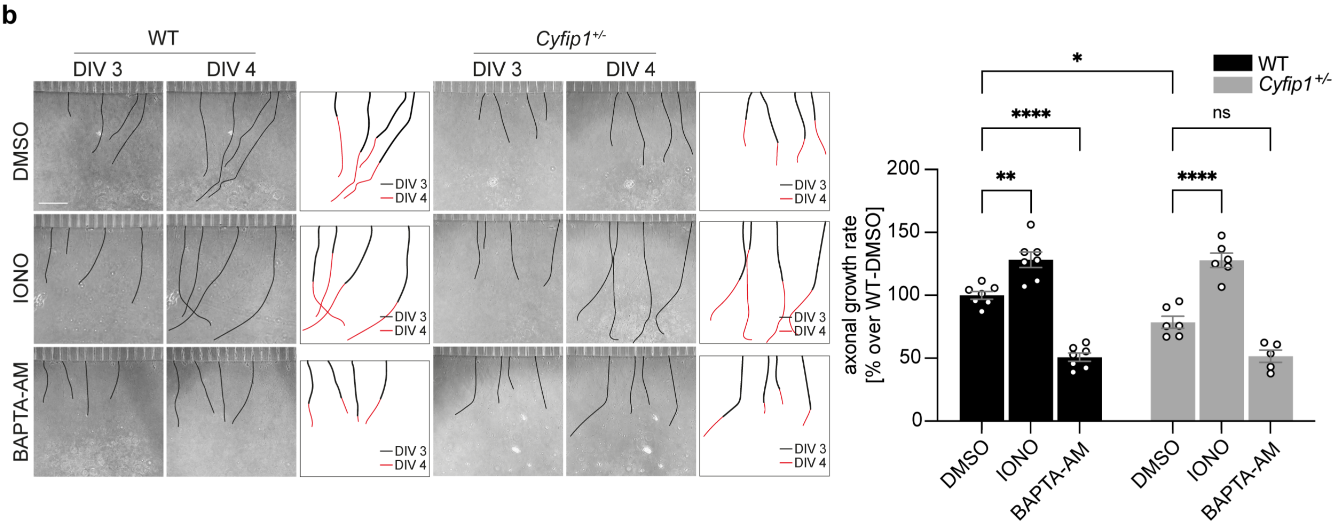

Fig. S5. Manipulation of intracellular Ca^{2+} levels impacts axonal growth. a Left, representative images of Fluo-4 AM intensity in the axons of WT and *Cyfip1*$^{+/-}$ DIV 3 cortical neurons after DMSO, Ionomycin (IONO) or BAPTA-AM treatment. Scale bar 30 µm. Right, axoplasmic calcium concentration [Ca^{2+}] (nM) measured using Fluo-4 AM imaging (WT n=5 embryos, *Cyfip1*$^{+/-}$ n=4 embryos; mean ± SEM; Two-Way ANOVA, $F_{(2,21)}$ = 3.996, p=0.0338; genotype effect p=0.0008, treatment effect p<0.0001, interaction p=0.0338). **b** Left, representative images of axons growing in the axonal compartment of microfluidic devices at DIV 3 and DIV 4 for WT and *Cyfip1*$^{+/-}$ neurons treated with DMSO, Ionomycin (1 µM), or BAPTA-AM (10 µM). Scale bar 200 µm. Growth rate of each individual axon was quantified as represented in the scheme (black line represents DIV 3 and red line DIV 4). Right, average axonal growth rate in WT and *Cyfip1*$^{+/-}$ neurons treated with DMSO, Ionomycin or BAPTA-AM (WT n=7 embryos, 149 DMSO-treated axons, 143 Ionomycin-treated axons and

135 BAPTA-AM treated axons, and *Cyfip1*$^{+/-}$ n=6 embryos, 121 DMSO-treated axons, 148 Ionomycin-treated axons and 101 BAPTA-AM treated axons; mean ± SEM; Two-Way ANOVA, $F_{(2,32)}$ = 3.473, p=0.0432; treatment effect p<0.0001, genotype effect p=0.081, interaction p=0.043). Source data are provided as a Source Data file.

Table S1

	Gene	Technique	Species	Forward Primer Sequence (5' to 3')	Reverse Primer Sequence (5' to 3')	Product Size (bp)	Tm (°C)	Accession Number
1	*mMtap1B*	RT-qPCR	*Mus musculus*	TTCCAGGACAAAAGATTCTTC	GGCTTCATCTGAAGGGTTGA	77	53.72/57.50	NM_008634.2
2	*mHprt1*	RT-qPCR	*Mus musculus*	CAGCCCCAAAATGGTTAAGGTTGC	TCCAACAAAGTCTGGCCTGTATCC	82	63.13/62.63	NM_013556.2
3	*mH3f3b*	RT-qPCR	*Mus musculus*	ACTGACTGTTCACAGACC	CCATCCCTTCTGCGTATTAG	77	51.50/52.60	NM_008211.3
4	*mCacna1c*	RT-qPCR	*Mus musculus*	TCCTCATCGTCATTGGGAGC	AGATGCGGGAGTTCTCCTCT	108	59.53/60.03	NM_009781.4
5	*mCacna1e*	RT-qPCR	*Mus musculus*	GCTACCGTGCCTGGATAGAC	CAAGGCTGATGTTCCCGAGT	77	59.53/59.32	NM_009782.3
6	*mCacna1i*	RT-qPCR	*Mus musculus*	GATTGCACTTCAGGGGTCCT	TGGCATCCAAAGACGTGTCA	98	59.67/59.89	NM_001044308.2
7	*mCacng2*	RT-qPCR	*Mus musculus*	TCTGTGCAAGCAAATCGACC	GATCGGGAAGATACTCGAGGC	103	59.13/59.80	NM_007583.2
8	*mCacnb3*	RT-qPCR	*Mus musculus*	CCGCTAACCCGGTCTATGTC	GTGTAGGAGTCGGCTGAACC	77	59.97/60.11	NM_001044741.3
9	*mRpS23*	RT-qPCR	*Mus musculus*	TGGTCGAAAAGGTCATGCTG	TGGCCTTTCTTTCTTGCCTTTG	109	58.48/59.90	NM_024175.3
10	*mMt12s*	RT-qPCR	*Mus musculus*	ACTCAAAGGACTTGGCGGTA	GAGATGGTGAGGTAGAGCGG	83	58.95/59.33	NC_005089.1

Table S1. **RT-qPCR primer sequences.** Table listing the primer sequences used for each gene analyzed by RT-qPCR.
