# Supplementary material for: CYFIP1 governs the development of cortical axons by modulating calcium availability
Source: Nat Commun. 2025 Nov 28;16:10764. doi: 10.1038/s41467-025-65801-0 (PMC12663352; doi:10.1038/s41467-025-65801-0)
Supplement: Supplementary file 2 — Reporting Summary [file 41467_2025_65801_MOESM2_ESM.pdf]

nature portfolio

Corresponding author(s): Claudia Bagni

Last updated by author(s): Sep 1, 2025

Reporting Summary

Nature Portfolio wishes to improve the reproducibility of the work that we publish. This form provides structure for consistency and transparency in reporting. For further information on Nature Portfolio policies, see our Editorial Policies and the Editorial Policy Checklist.

Statistics

For all statistical analyses, confirm that the following items are present in the figure legend, table legend, main text, or Methods section.

n/a	Confirmed	
☐	☒	The exact sample size (n) for each experimental group/condition, given as a discrete number and unit of measurement
☐	☒	A statement on whether measurements were taken from distinct samples or whether the same sample was measured repeatedly
☐	☒	The statistical test(s) used AND whether they are one- or two-sided<br>*Only common tests should be described solely by name; describe more complex techniques in the Methods section.*
☐	☒	A description of all covariates tested
☐	☒	A description of any assumptions or corrections, such as tests of normality and adjustment for multiple comparisons
☐	☒	A full description of the statistical parameters including central tendency (e.g. means) or other basic estimates (e.g. regression coefficient) AND variation (e.g. standard deviation) or associated estimates of uncertainty (e.g. confidence intervals)
☐	☒	For null hypothesis testing, the test statistic (e.g. F, t, r) with confidence intervals, effect sizes, degrees of freedom and P value noted<br>*Give P values as exact values whenever suitable.*
☒	☐	For Bayesian analysis, information on the choice of priors and Markov chain Monte Carlo settings
☒	☐	For hierarchical and complex designs, identification of the appropriate level for tests and full reporting of outcomes
☒	☐	Estimates of effect sizes (e.g. Cohen's d, Pearson's r), indicating how they were calculated

Our web collection on statistics for biologists contains articles on many of the points above.

Software and code

Policy information about availability of computer code

Data collection	Detailed description can be found in the material and methods section. In brief, images were acquired using the microscope specific software (LAS for all Leica microscopes), RT-qPCR was performed on StepOnePlus™ Real-Time PCR System (Thermo Fisher scientific), Western Blots were acquired using the LAS-4000 mini imaging system (GE Healthcare), and ATP levels were measured using Varioskan™ LUX Multimode Microplate Reader (Thermo Fisher scientific) equipped with SkanIt software (version 7.0.1).
Data analysis	Detailed description can be found in the material and methods section. In brief: image analysis was performed using Fiji (ImageJ) (version 1.49 and 2.16.0, NIH, USA). Kymographs were analyzed with Kymolyzer (Basu et al., 2020). Western blot quantification was performed using the ImageQuant software (GE Healtcare).The frequency of HuD and HuR binding sites on calcium channel subunits mRNAs has been predicted using RBPmap software and graphs showing the predicted frequency of binding sites were generated using R 4.1. Confocal image aquisition was performed using a LAS-X 3.0 software.

For manuscripts utilizing custom algorithms or software that are central to the research but not yet described in published literature, software must be made available to editors and reviewers. We strongly encourage code deposition in a community repository (e.g. GitHub). See the Nature Portfolio guidelines for submitting code & software for further information.

Data

Policy information about availability of data

All manuscripts must include a data availability statement. This statement should provide the following information, where applicable:

- Accession codes, unique identifiers, or web links for publicly available datasets
- A description of any restrictions on data availability
- For clinical datasets or third party data, please ensure that the statement adheres to our policy

Raw data is provided as a source data file

Research involving human participants, their data, or biological material

Policy information about studies with human participants or human data. See also policy information about sex, gender (identity/presentation), and sexual orientation and race, ethnicity and racism.

Reporting on sex and gender	N/A
Reporting on race, ethnicity, or other socially relevant groupings	N/A
Population characteristics	N/A
Recruitment	N/A
Ethics oversight	N/A

Note that full information on the approval of the study protocol must also be provided in the manuscript.

Field-specific reporting

Please select the one below that is the best fit for your research. If you are not sure, read the appropriate sections before making your selection.

☒ Life sciences ☐ Behavioural & social sciences ☐ Ecological, evolutionary & environmental sciences

For a reference copy of the document with all sections, see nature.com/documents/nr-reporting-summary-flat.pdf

Life sciences study design

All studies must disclose on these points even when the disclosure is negative.

Sample size	Sample size was determined based on previous literature in the field (Dominguez-Iturza et al., 2019; De Rubeis et al., 2013) and preliminary data from our laboratory.
Data exclusions	No data was excluded from the analysis.
Replication	All experiments were performed from several independent biological samples across different days, as mentioned in each figure legend. Axonal growth in vitro, mitochondrial motiliy and TMRE analysis were performed and/or analyzed by independent researchers. Attempts for replication were all successful.
Randomization	Samples were allocated based on the corresponding genotype. No randomization was required for our experiments.
Blinding	For most of the experiments the investigators were blind to the genotype during data collecting and analysis.

Reporting for specific materials, systems and methods

We require information from authors about some types of materials, experimental systems and methods used in many studies. Here, indicate whether each material, system or method listed is relevant to your study. If you are not sure if a list item applies to your research, read the appropriate section before selecting a response.

Materials & experimental systems

n/a	Involved in the study	
☐	☒	Antibodies
☒	☐	Eukaryotic cell lines
☒	☐	Palaeontology and archaeology
☐	☒	Animals and other organisms
☒	☐	Clinical data
☒	☐	Dual use research of concern
☒	☐	Plants

Methods

n/a	Involved in the study	
☒	☐	ChIP-seq
☒	☐	Flow cytometry
☒	☐	MRI-based neuroimaging

Antibodies

Antibodies used

Rabbit anti-CYFIP1, Millipore (Sigma-Aldrich) (Cat#AB6046); Mouse anti-bTubulinIII, 1:200, BioLegend (Cat#801201); Rabbit anti-CaV1.2 (CACNA1C), 1:100, Alomone Labs (Cat#ACC003); Rabbit anti-CaV2.3 (CACNA1E), 1:100, Alomone Labs (Cat#ACC006); Rabbit anti-CaV3.3 (CACNA1I), 1:100, Alomone Labs (Cat#ACC009); Rabbit anti-CaVb3 (CACNB3), Alomone Labs (Cat#ACC008); Rabbit anti-CaVg2 (Stargazin/CACNG2), Alomone Labs (Cat#ACC012); Alexa Fluor 488 goat anti-mouse IgG, Life Technologies (Cat#A11029); Alexa Fluor 555 goat anti rabbit IgG, Life Technologies(Cat#A21428); Anti-mouse IgG, HRP-linked Antibody, Cell Signaling Technology (Cat#7076S); Anti-rabbit IgG, HRP-linked Antibody, Cell Signaling Technology (Cat#7074S), mouse anti-HuD (H9), Santa Cruz (#SC-48421), mouse anti-HuR (3A2) - Santa Cruz (#SC-5261).

Validation

All antibodies used in this study have been previously reported in publications by other researchers (see manufactures web page for more information).

Animals and other research organisms

Policy information about studies involving animals; ARRIVE guidelines recommended for reporting animal research, and Sex and Gender in Research

Laboratory animals

Detailed description can be found in the material and methods section. In brief, the Cyfip1+/- mouse line, generated by gene trap at the Sanger Institute, UK, was kindly provided by Seth G.N. Grant. The gene trap cassette was inserted between exon 12 and 13. Molecular and behavioral characterization of the Cyfip1+/- mice was previously described in Dominguez-Iturza et al., 2019; De Rubeis et al., 2013. C57/Bl6 wild type (WT) and Cyfip1 heterozygous (Cyfip1+/-) mice were used at embryonic and postnatal days (E15.5, P5, P15, P30).

Wild animals

N/A

Reporting on sex

Cyfip1+/- male were crossed to wild-type C57Bl6 females and both male and females mice were used for the analysis.

Field-collected samples

N/A

Ethics oversight

Animal housing and care was conducted according to the institutional guidelines that are in compliance with national and international laws and policies (European Directive 2010/63/EU on the protection of animals used for scientific purposes of 20 October 2010, Italian D.Lgs 26/2014, Swiss Loi fédérale sur la protection des animaux 455, and Belgian Royal Decree of 29 May 2013). Studies performed in Italy were approved by the Institutional Ethical Board at the University of Rome Tor Vergata, according to the Guideline of the Italian Institute of Health (protocol n. 745/2022-PR). All the experimental procedures performed in Switzerland complied with the Swiss National Institutional Guidelines on Animal Experimentation and were approved by the Cantonal Veterinary Office Committee for Animal Experimentation. In all cases special attention was given to the implementation of the 3 R's, housing and environmental conditions and analgesia to improve the animals' welfare. Animal manipulation performed in Belgium was done according to the ECD protocol approved by the Institutional ethical committee of the KU Leuven and Cantonal Veterinary Office Committee for Animal Experimentation.

Note that full information on the approval of the study protocol must also be provided in the manuscript.

Plants

Seed stocks

N/A

Novel plant genotypes

N/A

Authentication

N/A
